# Supplementary material for: Naked mole-rat mortality rates defy Gompertzian laws by not increasing with age
Source: eLife. 2018 Jan 24;7:e31157. doi: 10.7554/eLife.31157 (PMC5783610; doi:10.7554/eLife.31157)
Supplement: Transparent reporting form [file elife-31157-transrepform.docx]

***eLife’s* transparent reporting form**

We encourage authors to provide detailed information *within their submission* to facilitate the interpretation and replication of experiments. Authors can upload supporting documentation to indicate the use of appropriate reporting guidelines for health-related research (see [EQUATOR Network](http://www.equator-network.org/%20)), life science research (see the [BioSharing Information Resource](https://biosharing.org/)), or the [ARRIVE guidelines](http://www.plosbiology.org/article/info:doi/10.1371/journal.pbio.1000412) for reporting work involving animal research. Where applicable, authors should refer to any relevant reporting standards documents in this form.

If you have any questions, please consult our Journal Policies and/or contact us: [editorial@elifesciences.org](mailto:editorial@elifesciences.org).

**Sample-size estimation**

- You should state whether an appropriate sample size was computed when the study was being designed
- You should state the statistical method of sample size computation and any required assumptions
- If no explicit power analysis was used, you should describe how you decided what sample (replicate) size (number) to use

Please outline where this information can be found within the submission (e.g., sections or figure legends), or explain why this information doesn’t apply to your submission:

Analyses were performed retrospectively, on >30-years’-worth of collected data. We analyzed the statistical robustness of all results using metrics recommended by eLife, as well as by running simulations on some sub-populations; however, we had no opportunity to meaningfully modify the sample size. We analyzed the impact of sample size from our existing data set in the form of 95% confidence intervals for hazard (Figures 1, 2, 3) and in the form of simulations of populations with varying compositions (Figure 4).

**Replicates**

- You should report how often each experiment was performed
- You should include a definition of biological versus technical replication
- The data obtained should be provided and sufficient information should be provided to indicate the number of independent biological and/or technical replicates
- If you encountered any outliers, you should describe how these were handled
- Criteria for exclusion/inclusion of data should be clearly stated
- High-throughput sequence data should be uploaded before submission, with a private link for reviewers provided (these are available from both GEO and ArrayExpress)

Please outline where this information can be found within the submission (e.g., sections or figure legends), or explain why this information doesn’t apply to your submission:

These analyses were performed on a single, historical cohort of naked mole-rats in a well characterized colony. We applied multiple different inclusion/exclusion criteria, and have described each and presented the results for each (Figure 2 and its legend, the Results section “Inclusion of low-resolution birth/death records reinforced constant-hazard demography”, and Methods).

**Statistical reporting**

- Statistical analysis methods should be described and justified
- Raw data should be presented in figures whenever informative to do so (typically when N per group is less than 10)
- For each experiment, you should identify the statistical tests used, exact values of N, definitions of center, methods of multiple test correction, and dispersion and precision measures (e.g., mean, median, SD, SEM, confidence intervals; and, for the major substantive results, a measure of effect size (e.g., Pearson's r, Cohen's d)
- Report exact p-values wherever possible alongside the summary statistics and 95% confidence intervals. These should be reported for all key questions and not only when the p-value is less than 0.05.

Please outline where this information can be found within the submission (e.g., sections or figure legends), or explain why this information doesn’t apply to your submission:

As a primarily statistics-based paper, this statistical information & discussion of its justification is pervasive across nearly the entire manuscript and figures. N values are given as comprehensively as they can be in the figures, given the scale of the dataset. For any ambiguity that remains, a complete animal-by-animal annotated accounting is provided as Supplemental Data File 1.

(For large datasets, or papers with a very large number of statistical tests, you may upload a single table file with tests, Ns, etc., with reference to sections in the manuscript.)

**Group allocation**

- Indicate how samples were allocated into experimental groups (in the case of clinical studies, please specify allocation to treatment method); if randomization was used, please also state if restricted randomization was applied
- Indicate if masking was used during group allocation, data collection and/or data analysis

Please outline where this information can be found within the submission (e.g., sections or figure legends), or explain why this information doesn’t apply to your submission:

There were no experimental groups in this study. Animals were grouped in terms of biological/behavioral classifications (sex, breeding-status), but those categories represent observations rather than assignments. We describe the criteria for classification in the section “Non-increasing hazard was exhibited by breeders and non-breeders of both sexes” as well as in the Methods and provide our animal-by-animal classifications in Supplemental Data File 1.

**Additional data files (“source data”)**

- We encourage you to upload relevant additional data files, such as numerical data that are represented as a graph in a figure, or as a summary table
- Where provided, these should be in the most useful format, and they can be uploaded as “Source data” files linked to a main figure or table
- Include model definition files including the full list of parameters used
- Include code used for data analysis (e.g., R, MatLab)
- Avoid stating that data files are “available upon request”

Please indicate the figures or tables for which source data files have been provided:

We have uploaded the full source data (animal-by-animal annotation of birth, death/censorship, sex/breeding status, and other historical notes), annotated with inclusion/exclusion decisions for dates, as well as the original annotations used to make those decisions, as Supplemental Data File 1. The exact parameters for the simulations we ran to assess robustness (Figure 4) are provided as Supplemental Data File 2, and the outcomes of the simulations themselves are provided in Supplemental Data Files 3-6. A stand-alone python script that executes such simulations is provided as a Supplemental File as well.
